# Long-term survival of stage IV melanoma patients: evaluation on 640 melanoma patients entering stage IV between 2014 and 2017

**DOI:** 10.1007/s00432-023-05533-0

**Published:** 2024-01-18

**Authors:** Markus Reitmajer, Ulrike Leiter, Lena Nanz, Teresa Amaral, Lukas Flatz, Claus Garbe, Andrea Forschner

**Affiliations:** Department of Dermatology, University Hospital Tuebingen, Liebermeisterstraße 25, 72076 Tuebingen, Germany

**Keywords:** Melanoma stage IV, Survivorship, Long-term survivors, Immune checkpoint inhibitor (ICI), Targeted therapy (TT)

## Abstract

**Purpose:**

Since the introduction of immune checkpoint inhibitors (ICI) and targeted therapies (TT), survival rates of metastatic melanoma patients have increased significantly and complete remissions are no longer rarities. Consequently, there is an increasing number of long-term survivors who have not yet been comprehensively characterized.

**Methods:**

We included melanoma patients who entered stage IV between 2014 and 2017 and survived at least 5 years after entering stage IV. Descriptive statistics were performed to characterize the applied systemic therapies, response rates and to report which of these patients are still alive today.

**Results:**

640 patients entered stage IV at the University Hospital Tuebingen. Of these, 207 patients (32%) were still alive at least 5 years after entering stage IV. Details of applied therapies and response rates were available in 176 patients (85%). About 90% of patients (*n *= 159) were still alive at the time of analysis. Median survival since first stage IV diagnosis was 6.0 years (range 5–9 years). An impressive majority of patients (*n* = 146, 83%) were no longer receiving systemic therapy at the time of evaluation. Complete remission under first line systemic therapy was seen in 36% of the patients.

**Conclusion:**

This dataset comprises the largest available cohort of long-term surviving stage IV melanoma patients. Since 90% of patients in our cohort are still alive today, we expect an increasing number of long-term survivors in the future. Our data indicate the need for specific follow-up programs addressing the needs of long-term survivors.

**Supplementary Information:**

The online version contains supplementary material available at 10.1007/s00432-023-05533-0.

## Introduction

Until 2010, less than 5% of the patients with stage IV melanoma survived 5 years (Manola et al. 2000; Tsao et al. 2004; Balch et al. 2009). With the approval of the immune checkpoint inhibitors (ICI) ipilimumab in 2011 (Hodi et al. 2010; Tsao et al. 2004), nivolumab and pembrolizumab in 2015 and the combination of ipilimumab and nivolumab in 2016, overall survival (OS) increased markedly and complete remissions are no longer rarities (Balch et al. 2009; Rockberg et al. 2016; Tichanek et al. 2023; van Zeijl et al. 2021). For patients with BRAF^V600^ mutant melanoma, BRAF and MEK inhibitors offer likewise excellent treatment options (McArthur et al. 2014; Hauschild et al. 2012; Rogiers et al. 2019; Long et al. 2017, 2014).

These novel treatment options have significantly increased melanoma-specific survival (MSS) and we are now encountering an increasing percentage of long-term survivors in our outpatient departments who rarely existed before. It is known from other tumor entities, that some cancer survivors recover without constraints, while others suffer physical, psychological, financial or social impairments (Medicine and Council 2006; Stein et al. 2008). While survivorship programs have already been established as part of the clinical routine in other tumor entities such as breast cancer, prostate cancer, and lymphoma, it is uncommon in metastasized melanoma patients (Shapiro 2018; Rosenthal 2022; Pinto et al. 2022; Shrem et al. 2022). The term “long-term survivors” is often used for patients who were diagnosed more than five years ago. Epidemiological studies estimate that at least 4.5 million people in Germany are living with or after cancer and that around two-thirds of these cancer survivors can be considered long-term survivors (Arndt 2019).

Limited knowledge exists on the characteristics and applied systemic therapies of long-term surviving patients with stage IV melanoma in a real-world setting. However, this information is crucial to adapt care for the growing population of long-term survivors and to implement a survivorship approach (Medicine and Council 2006). In this retrospective single-center study, we aimed to characterize all melanoma patients diagnosed with stage IV between 2014 and 2017 who have survived for at least 5 years after entering stage IV. The primary focus of this study was to analyze the type of systemic therapies applied, including response rates, the type and localization of radiotherapy and to evaluate who among these patients is still alive today.

## Methods

### Study design

We used the institutional database from the central malignant melanoma registry (CMMR) to identify potentially eligible patients with stage IV entry between 01/01/2014 and 12/31/2017 which had been treated at the University Hospital Tuebingen and who were alive for at least 5 years after entering stage IV (Garbe et al. 1995; Leiter et al. 2004). We collected information on the type of systemic therapy, surgical procedures, and radiotherapy from the medical records. Tumor-specific data, as well as patients’ age, gender, date of initial diagnosis, and subsequent disease course were obtained directly from the CMMR. Follow-up time was defined as the time between entry in stage IV and the last contact. The data cut-off date was 04/01/2023. Systemic therapies were grouped as follows: targeted therapy (TT): BRAF or MEK inhibitors as monotherapy or combined BRAF and MEK inhibitors, Immune checkpoint inhibitors (ICI): ipilimumab, nivolumab or pembrolizumab as monotherapy or ipilimumab and nivolumab as combination, chemotherapy or study therapy, if it was not clear, in which treatment arm patients had been included, thus the specific treatment type could not be classified to either TT, ICI or chemotherapy. Response to the systemic therapy was classified according to the revised response evaluation criteria in solid tumors (RECIST) guidelines (version 1.1) with complete response (CR), partial response (PR), stable disease (SD), or progressive disease (PD) as possible outcomes. Patients with a mixed response, for example increasing and decreasing lung metastases in the same scan were grouped as SD. Overall response rate (ORR) was defined as the sum of CR and PR. Disease control rate (DCR) was defined as the sum of CR, PR and SD.

All patients included in the CMMR provided written informed consent for documentation of their clinical data for research purposes and publications. This retrospective analysis adhered to the guidelines of the local ethical committee of the University Hospital Tübingen and followed the general recommendations outlined in the Declaration of Helsinki.

### Statistical analysis

Demographic and clinical data were characterized using statistical descriptive analyses conducted with IBM^®^ SPSS^®^ Statistics 28.0.0.0 (IBM, Armonk, USA). Graphs were generated using GraphPad PRISM^®^ 9.5.0 (Dotmatics, Boston, USA).

## Results

### Patient characteristics

Between 01/01/2014 and 12/31/2017, 640 melanoma patients entered stage IV at the University Hospital Tuebingen. A total of 207 patients (32%) were still alive at least 5 years after entering stage IV. Details of therapies of these long-term survivors were available in 176 patients (85%), who could thus be included in the retrospective analysis. 44% of the included patients were female and 56% male. The median age of patients at the time of entering stage IV was 62.5 years, ranging from 25 to 85 years. The time between stage IV entry and the last contact was 6.0 years in median, ranging from 5 to 9 years. Between initial diagnosis and entering stage IV in median 2.0 years passed, ranging from 0 to 30 years (Table 1).

**Table 1:** Baseline patient characteristics total cohort

	*N*	%
Melanoma patients stage IV between 01/01/2014 and 12/31/2017	640	
Alive at least 5 years after entering stage IV	207	
Alive at least 5 years after entering stage IV and available details about therapies (= cohort)	176	100
Sex		
Female	77	44
Male	99	56
Years between initial diagnosis and entry into stage IV Median [range]	2	[0–30]
Age at the time of stage IV diagnosis Median [range]	62.5	[25–85]
Years between entry into stage IV and last contact Median [range]	6.0	[5–9]
AJCC stage at primary diagnosis		
Stage IA–B	36	21
Stage IIA–C	51	29
Stage IIIA–D	55	31
Stage IV	20	11
Unknown	14	8
Melanoma type		
Cutaneous	136	77
Unknown primary	18	10
Acral lentiginous	11	6
Uveal	8	5
Mucosal	3	2
Metastasis to distant organs		
Lung	100	57
Liver	54	31
Central nervous system	42	24
BRAF^V600^ mutation		
Wildtype	90	51
Mutant	72	41
Unknown	14	8
Alive at the time of the survey (04/01/2023)	159	90
Still receiving systemic therapy at the time of the survey (04/01/2023)	21	12
ICI at any time point		
Yes	129	73
No	47	27
TT at any time point		
Yes	38	22
No	138	78
Systemic therapies for metastases		
One	146	83
Two	73	41
Three or more	30	17
No systemic therapy for metastases (*n* = 29)	29	16
Adjuvant systemic therapy only	11	6
Radiotherapy only	4	2
Surgery/stereotaxy/ radiofrequency ablation only	14	8
Radiotherapy at any time point?		
Yes	70	40
No	106	60
Radiotherapy		
Brain	24	14
Soft tissue/regional lymph nodes	36	21

Most of the patients (*n* = 136, 77%), had the histological type of cutaneous melanoma. The second most common subtype consisted of patients with occult melanoma, accounting for 10% of the total cohort (Table 1). Pulmonary metastasis occurred in 57% (*n* = 100) of patients. 31% (*n* = 54) of patients had liver metastasis, and 24% (*n* = 42) had at least one brain metastasis. Mutational analysis from tumor tissue was available in 92% (*n* = 162) of the cases. Among the analyzed cases, 41% (*n* = 72) of patients had a BRAF^V600^ mutation. NRAS mutation was detected in 18% of the cases (*n* = 31).

At the time of evaluation, the majority of the cohort (*n* = 159, 90%) was still alive. Some patients had considerably surpassed 5-years survival and only 12% (*n* = 21) of the patients were still receiving systemic therapy (Table 1).

### Characterization of the applied systemic therapies, radiotherapy and surgery

**Fig. 1:**
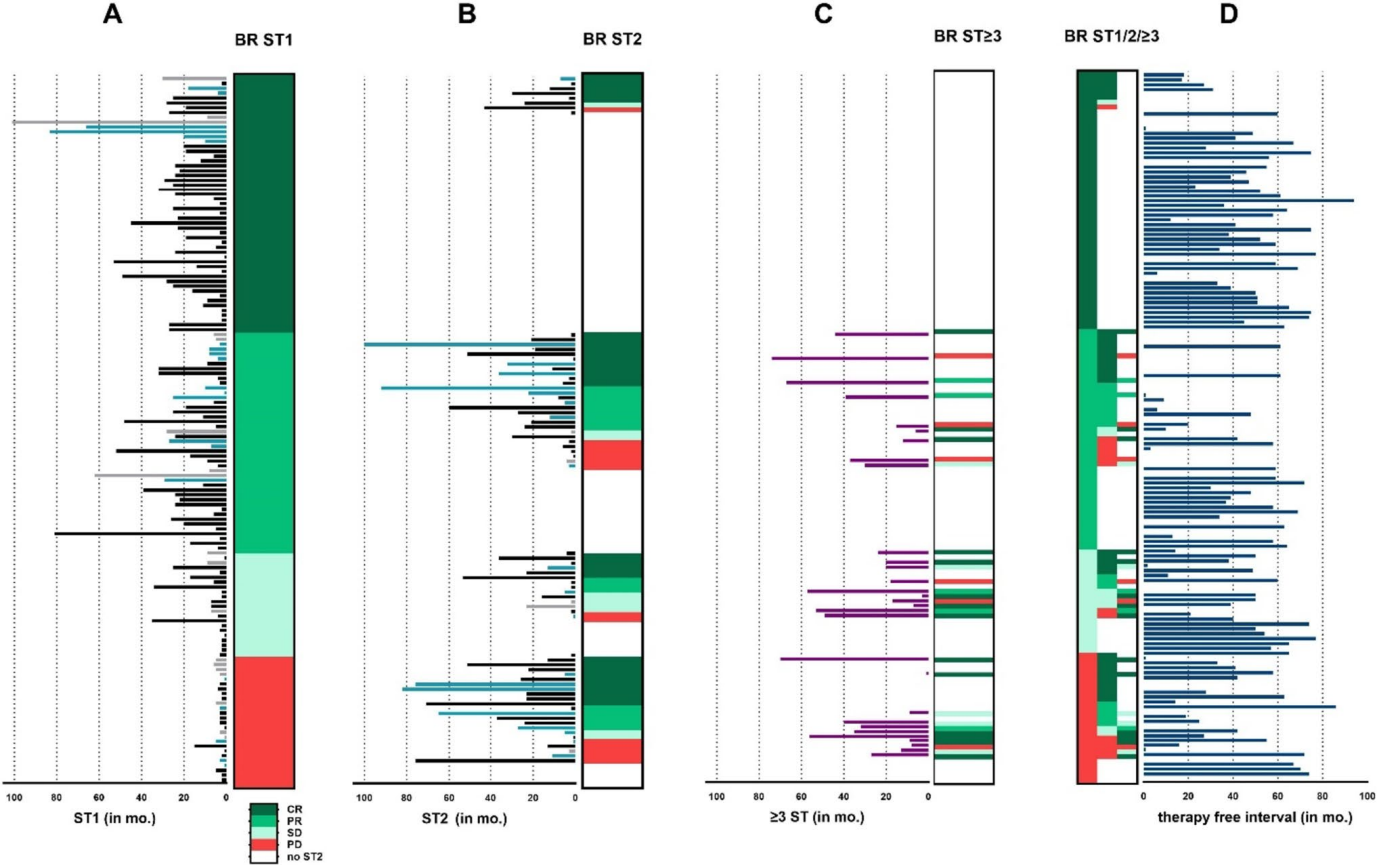
Best response and duration of the applied therapies and illustration of the therapy-free survival. Graph 1 displays in horizontal lines the respective duration of systemic therapy (ST) 1, 2 or ≥ 3 in the metastatic setting. The color bars of the duration lines (in months (mo.)) are as follows: black for IT, light blue for TT and grey for chemotherapy or study therapy. The respective best response (BR) is displayed in the columns with the following color coding, Complete response (CR): dark green, partial response (PR): middle green, stable disease (SD): light green, progressive disease (PD): red. If no ST had been applied, the column is white. Graph 1**A**: first systemic therapy (ST1). Graph 1**B**: second systemic therapy (ST2). Graph 1**C**: if more than 3 ST (ST ≥ 3) had been applied, duration line is displayed cumulative (violet bars) and only the BR of all ≥ 3 responses is displayed. Graph 1**D** shows the therapy-free interval after ending the last ST, represented by dark blue lines in months (mo.). The column summarizes BR of graphs 1(**A**–**C**)

The types of therapies applied and responses are summarized in Table 2. Out of 176 patients in total, 146 (83%) had received at least one systemic therapy in the metastatic setting (Fig. 1). All of these patients had inoperable metastases. Among these 146 patients, 73 required at least one additional, second-line systemic therapy and 17% of the patients (*n* = 30) had three or more systemic therapies in the metastatic setting.

**Table 2:** Best response and type of therapy

Type	Best response first systemic therapy				Total
	CR	PR	SD	PD	
ICI					
Ipilimumab	7	1	5	3	16 [11%]
Nivolumab or pembrolizumab	21	11	6	7	45 [31%]
Combined ipilimumab and nivolumab	16	18	7	5	46 [32%]
TT					
BRAF or MEK inhibitor monotherapy	1	4	0	3	8 [5%]
Combined BRAF and MEK inhibitor	5	6	0	2	13 [9%]
Other					
Chemotherapy	2	1	1	6	10 [7%]
Study	1	4	2	1	8 [5%]
Total *n* [%]	53 [36%]	45 [31%]	21 [14%]	27 [18%]	146 [100%]

Type	Best response second systemic therapy					Total
	CR	PR	SD	PD	No data	
ICI						
Ipilimumab	1	2	0	1	0	4 [5%]
Nivolumab or pembrolizumab	19	6	3	2	0	30 [41%]
Combined ipilimumab and nivolumab	4	4	0	5	1	14 [19%]
TT						
BRAF or MEK inhibitor monotherapy	0	0	2	1	0	3 [4%]
Combined BRAF and MEK inhibitor	8	5	1	3	0	17 [23%]
Other						
Chemotherapy	0	0	2	1	0	3 [4%]
Study	0	0	1	1	0	2 [3%]
Total *n* [%]	32 [44%]	17 [23%]	9 [12%]	14 [19%]	1 [1%]	73[100%]

Almost three quarters of the patients (*n* = 129, 73%) received ICI in the metastatic setting at any time point, but only 22% of the patients had at least one course of TT in the metastatic setting. Additionally, 40% of the patients (*n* = 70) received at least one course of radiotherapy either in an adjuvant or a metastatic setting. Among those treated by radiotherapy, more than one third (*n* = 24) had received radiotherapy of brain metastases and more than 50% (*n* = 36) for soft tissue or regional lymph nodes metastases (Table 1).

Only 29 patients had never received any systemic therapy in the metastatic setting. Out of these patients, 14 patients underwent solely local ablative procedures for metastases, such as surgery, radiofrequency ablation or stereotactic radiotherapy (Table 1).

Table 2 presents the best response rates of respective types of therapies. The upper section shows the data for the first systemic therapy in the metastatic setting, while the lower section of the table shows the data for the second systemic therapy. In the case of one patient, the best response could not be determined. The following abbreviations were used: ICI (immune checkpoint inhibitors), TT (targeted therapy), CTX (chemotherapy), Study (study medication), CR (complete response), PR (partial response), SD (stable disease), PD (progressive disease).

Table 3 shows the best response with the first systemic therapy (ST1) in the group of patients who received a second systemic therapy in the metastatic setting. In the case of one patient, the best response could not be determined. Other abbreviations used are as follows: CR (complete response), PR (partial response), stable disease (SD), or progressive disease (PD).

**Table 3:** Best response in ST2

Response ST 1	Response second systemic therapy (ST2)					
	CR	PR	SD	PD	No data	Total
CR	6	0	1	1	0	8 [11%]
PR	11	9	2	6	0	28 [38%]
SD	5	3	4	2	0	14 [19%]
PD	10	5	2	5	1	23 [32%]
Total [%]	32 [44%]	17 [23%]	9 [12%]	14 [19%]	1 [1%]	73 [100%]

### Analysis of best response rates to systemic therapies

The overall response rate (ORR) was 67% in both, the first and the second systemic therapy line (Table 2, Fig. 1). It is remarkable that the CR rate was 36% in the first-line and 44% in the second-line cohort. Table 3 shows the best response to the second-line therapy depending on the outcome of the first-line therapy. In the supplement, further details on the applied second-line therapies and the corresponding first-line therapies and their responses are displayed.

ICI was the most common type of systemic therapy for both, the first-line (72%) and the second-line therapy (66%). On the other hand, TT had been applied more often in the second-line therapy. In the first-line cohort, 16% of patients received TT, while in the second-line cohort, 27% received TT.

## Discussion

With the introduction of ICI and TT, the treatment landscape for stage IV melanoma has undergone a significant improvement (Tsao et al. 2004; Keung and Gershenwald 2018; Wolchok et al. 2022). Consequently, we are now observing an increasing group of long-term survivors.

A large proportion of our long-term surviving patients with stage IV melanoma had received ICI at any time point during their disease (73%). Notably, the vast majority of the cohort (*n* = 159, 90%) was still alive at the time of the evaluation and in most of them, systemic therapy could be stopped in the meantime. It is important to consider that when looking at long-term survivors of stage IV melanoma, of course a high percentage of patients must have achieved a very good treatment response, otherwise they would probably not have been long-term survivors. When looking at the type of therapies applied, it is obvious that the relevance of ICI is extremely high. Not only by combined ICI with ipilimumab and nivolumab, but also by monotherapy with nivolumab or pembrolizumab, ORRs of 74% and 71% could be achieved. Considering the lower risk of suffering grade 3 or 4 immune-related side effects, it is therefore worth looking at who needs combined ICI and for whom monotherapy may be sufficient. The question of where PD-1 antibodies as monotherapy might be sufficient and where not is certainly not easy to answer. Combination ICI therapy appears to improve survival rates for patients with BRAF-mutated tumors, asymptomatic brain metastases or PD-L1-negative status compared to PD-1 antibodies as monotherapy (Wolchok et al. 2022).

Only a few patients (*n* = 21) of our cohort had TT as first-line treatment and half of them (*n* = 11) received ICI as second-line therapy. However, the frequency of BRAF-mutated tumors was only 41%, thus lower than the reported which might be explained by the fact that melanoma patients with BRAF mutations have a worse prognosis, thus a higher risk of mortality compared to patients without BRAF mutations (Davies et al. 2002; Banerji et al. 2008; Edlundh-Rose et al. 2006; Safaee Ardekani et al. 2012).

Considering the data of the “DREAMseq” and “SECOMBIT” trials, patients with first-line TT and switch to combined ICI only in case of progression, had worse overall survival compared to patients with combined ICI first-line (Ascierto et al. 2023; Atkins et al. 2023). These recently published data on the optimal sequencing of TT and ICI therapy underline our observation that most of the long-term survivors had ICI as first-line treatment. Nevertheless, it has to be considered that in our cohort the switch to ICI after progression with TT was obviously also a successful option. However, there are hints that resistance mechanisms towards BRAF and MEK inhibitors might cause cross-resistance towards ICI (Haas et al. 2021). Subgroup analyses revealed that patients with 3 or more metastatic involved organs or elevated LDH baseline had a worse outcome with TT as first-line therapy (Patel et al. 2023). This could be an additional hint to be considered when deciding on first-line therapies.

Currently, there is no established survivorship program for melanoma patients, despite the growing number of long-term surviving patients. In general, long-term survivors are often considered being cured, but they are often not healthy. In other cancer entities, it has already been reported, that long-term survivors form a special population with specific complaints that need to be addressed (Seifart 2022; DeSantis et al. 2014; Couey et al. 2019; Schulz et al. 2022; Johnson et al. 2022; Owen et al. 2021).

The high number of long-term survivors with a history of radiotherapy in our cohort underline the importance of an interdisciplinary therapeutic approach in the management of stage IV melanoma. With the availability of highly effective systemic therapies, also in the metastatic setting, the focus on subsequent harm must be considered from the beginning. Patients now have a high risk of experiencing the full effects of long-term toxicity, in former times, most of the patients had died before. Therefore, the treatment concept in stage IV melanoma patients should consider the high chance of complete remissions from the outset. This precautionary approach requires a team of dermatologists, surgeons, radiologists, and radiotherapists working together to optimize patient outcomes with the lowest possible risk of long-term toxicity. In a recently published study of long-term survivors under ICI in unresectable stages III and IV who survived more than 12 months, patients reported fatigue (28%), aching joints (17%) and aching muscles (12%) as the most frequent symptoms experienced (Mamoor et al. 2020). In another study on long-term surviving melanoma patients after ICI, fatigue was the most common mentioned complaint (Mamoor et al. 2020). Another prospective study found that over 50% of metastatic melanoma patients reported psychological distress baseline to ICI therapy. Despite decreasing values, the psycho-oncological burden during the course of the therapy remained considerably high (Wiens et al. 2021). In addition, an increased prevalence of psychological distress especially among younger long-term survivors has recently been reported (Abdelhadi 2023). All of these findings highlight the importance of psycho-oncological support to address distress and interdisciplinary care to manage side effects such as joint and muscle pain or endocrinological disorders.

To our knowledge, this is the largest dataset available that characterizes melanoma stage IV survivors who have surpassed the 5-year survival mark. One of the strengths of our study is certainly that the patients had been treated and documented according to the same standards of care at one single center. On the other hand, single-center studies always carry the risk of a selection bias. Furthermore, the care at an university hospital with close monitoring possibilities and follow-up may not necessarily represent the “real life” situation of stage IV melanoma patients. Our next step will be to assess the specific concerns and needs of these long-term survivors by questionnaires to provide appropriate support during the transition from the phase of active disease and acute medical treatment to the phase of long-term survival, back to “normal” life (Medicine and Council 2006). By gaining a deeper understanding of complaints and challenges, we might be able to develop follow-up care strategies tailored to the unique circumstances of long-term surviving melanoma patients.

## Supplementary Information

Below is the link to the electronic supplementary material.

Supplementary file1 (DOCX 12 kb)

## Data Availability

The datasets used and/or analyzed during the current study are available from the corresponding author upon reasonable request.
