# Supplementary material for: Long-term survival of stage IV melanoma patients: evaluation on 640 melanoma patients entering stage IV between 2014 and 2017
Source: J Cancer Res Clin Oncol. 2024 Jan 18;150(1):15. doi: 10.1007/s00432-023-05533-0 (PMC10796594; doi:10.1007/s00432-023-05533-0)
Supplement: Supplementary file 1 — Supplementary file1 (DOCX 12 kb) [file 432_2023_5533_MOESM1_ESM.docx]

**Supplement Table 1 ICI or TT as ST1 and ICI or TT as ST2**

| ICI or TT as ST1 | ICI or TT as ST2 | | |  |
| --- | --- | --- | --- | --- |
|  | TT | ICI mono | ICI combi | Total |
| TT | 4 | 9 | 2 | 15 [27%] |
| ICI mono | 8 | 16 | 8 | 32 [58%] |
| ICI combi | 5 | 3 | 0 | 8 [15%] |
| Total [%] | 17 [31%] | 28 [51%] | 10 [18%] | 55 [100%] |
